# c-FLIP links mTORC2 to apoptosis

**DOI:** 10.18632/oncoscience.40

**Published:** 2014-05-20

**Authors:** Liqun Zhao, Shi-Yong Sun

**Affiliations:** Department of Hematology and Medical Oncology, Emory University School of Medicine and Winship Cancer Institute, Atlanta, GA, USA

Cellular FLICE inhibitory protein (c-FLIP), a truncated form of caspase-8 that lacks caspase enzymatic activity, primarily acts as a specific inhibitor of the extrinsic death receptor-mediated apoptotic pathway [1]. Typically, a death ligand (e.g., TRAIL) binds to its corresponding death receptor (e.g., DR5) to induce oligomerization of the receptors. This will lead to recruitment of the adaptor molecule, FADD, and the initiator caspase, pro-caspase-8, to form a death inducing-signaling complex (DISC). In the DISC, pro-caspase-8 undergoes autocleavage and activation. The active caspase-8 further activates effector caspases that lead to apoptotic death. c-FLIP competes with pro-caspase-8 for binding to FADD, thereby suppressing DISC formation and caspase-8 activation. Many studies have shown that elevated c-FLIP expression protects cells from death receptor-mediated apoptosis, whereas downregulation of c-FLIP by chemicals or siRNA sensitizes cells to death receptor-induced apoptosis. Therefore, c-FLIP is a critical factor that affects cancer cell sensitivity to induction of apoptosis. Some small molecules that reduce c-FLIP level exhibit therapeutic potential with regard to sensitizing cancer cells to death receptor-induced apoptosis [1].

c-FLIP has multiple splice variants at the mRNA level, but only the long isoform (FLIP_L_) and short isoform (FLIP_S_) are detectable at the protein level in various types of cells and have been extensively studied. Both proteins exhibit anti-apoptotic ability in the DISC. It is well-known that both FLIP_L_ and FLIPs undergo ubiquitination/proteasome-dependent degradation and thus are rapidly turned over proteins with short half-lives [1]. However, the mechanisms underlying c-FLIP degradation are largely unknown even though some ubiquitin E3 ligases such as Itch and Cbl are reported to be involved in this process [1].

The mammalian target of rapamycin (mTOR) plays a critical role in the positive regulation of cell proliferation and survival, largely via forming two multi-protein complexes characterized by the essential partner protein raptor (forming mTOR complex 1; mTORC1) and rictor (forming mTOR complex 2; mTORC2) [2]. mTORC1 is sensitive to rapamycin and deeply involved in many key cellular processes to maintain cell metabolism and growth through regulation of protein synthesis by phosphorylating two key proteins, p70 S6 kinase and eIF4E binding protein 1 (4E-BP1) to facilitate the formation of the translation initiation complex. In contrast, mTORC2 is generally thought to be insensitive to rapamycin and its biological functions, particularly those related to cancer, are largely unknown [3].

In our recent publication [4], we found that one representative mTOR kinase inhibitor, PP242, effectively decreased FLIP_S_ levels and synergistically enhanced TRAIL-induced apoptosis in cancer cells although it alone had a very weak apoptosis-inducing activity. When ectopic FLIP_S_ was expressed, the synergistic apoptosis-inducing effects of the PP242 and TRAIL combination were abrogated, suggesting that FLIP_S_ downregulation accounts for the enhancement of TRAIL-induced apoptosis by PP242. To determine whether the effect of PP242 on decreasing FLIP_S_ is indeed due to inhibition of mTOR, we further tested other mTOR inhibitors and found that both INK128 (an mTOR kinase inhibitor) and BEZ235 (an PI3K/mTOR dual inhibitor), but not rapamycin (an allosteric mTOR inhibitor), decreased FLIP_S_ levels and enhanced TRAIL-induced killing of cancer cells. Moreover, we found that knockdown of rictor, but not raptor, with both siRNA and shRNA, mimicked the effects of PP242 on reducing FLIP_S_ levels and sensitized cancer cells to TRAIL-induced apoptosis. Collectively we believe that it is the mTORC2 inhibition that leads to FLIP_S_ downregulation and subsequent enhancement of TRAIL-induced apoptosis. Hence our findings provide the first evidence suggesting that mTORC2 regulates TRAIL induced apoptosis, likely through stabilizing FLIP_S_ expression.

The subsequent question is how the mTORC2 regulates FLIP_S_ expression. In this regard, we found that both PP242 treatment and rictor knockdown increased FLIP_S_ ubiquitination and promoted its degradation. Moreover, inhibition of the proteasome with MG132 rescued FLIP_S_ reduction induced by PP242. Complementarily, the enforced expression of rictor increased FLIP_S_ stability. These data together clearly show that mTORC2 stabilizes FLIP_S_ through suppressing ubiquitin/proteasome-mediated protein degradation. Inspired by a previous study suggesting that the E3 ubiquitin ligase, Cbl, acts to mediate FLIP_S_ ubiquitination and degradation [5], we further examined whether Cbl is involved in mTORC2 inhibition-induced FLIP_S_ degradation. We found that inhibition of Cbl by knocking down its expression not only elevated basal levels of FLIP_S_, but also prevented FLIP_S_ reduction induced by both PP242 and rictor knockdown. Hence, mTORC2 inhibition clearly induces Cbl-mediated FLIP_S_ degradation. Accordingly, we suggest that mTORC2 stabilizes FLIP_S_ protein by suppressing Cbl-mediated FLIP_S_ degradation.

In this study, we have not been able to address how mTORC2 negatively regulates Cbl-mediated FLIP_S_ degradation. It is known that the mTORC2 functions as an Akt S473 kinase to activate Akt signaling [3]. Some studies have revealed that Akt is involved in the positive regulation of c-FLIP expression, likely at transcriptional levels [6-8]. Our preliminary results suggest that mTORC2 inhibition-induced FLIP_S_ degradation is unlikely to be the consequence of Akt inhibition. Therefore further study is warranted to fully uncover the mechanism by which mTORC2 negatively regulates Cbl-mediated FLIP_S_ degradation.

Nonetheless, our findings suggest that mTORC2 negatively regulates the extrinsic death receptor-mediated apoptotic pathway by stabilizing FLIP_S_ via suppressing Cbl-mediated protein degradation, hence for the first time connecting mTORC2 to the regulation of death receptor-induced apoptosis. Accordingly, inhibition of mTORC2 (e.g., with mTOR kinase inhibitors) will down-regulate FLIP_S_ levels and sensitize cancer cells to undergo death receptor-induced apoptosis. Thus our findings suggest not only novel biological functions of the mTORC2 in the regulation of protein stability and apoptosis, but also a novel therapeutic strategy to enhance death receptor-targeted cancer therapy by the suppression of mTORC2.
